# Bright Fluorescence Monitoring System Utilizing *Zoanthus* sp. Green Fluorescent Protein (*ZsGreen*) for Human G-Protein-Coupled Receptor Signaling in Microbial Yeast Cells

**DOI:** 10.1371/journal.pone.0082237

**Published:** 2013-12-05

**Authors:** Yasuyuki Nakamura, Jun Ishii, Akihiko Kondo

**Affiliations:** 1 Department of Chemical Science and Engineering, Graduate School of Engineering, Kobe University, Kobe, Japan; 2 Organization of Advanced Science and Technology, Kobe University, Kobe, Japan; University of Oldenburg, Germany

## Abstract

G-protein-coupled receptors (GPCRs) are currently the most important pharmaceutical targets for drug discovery because they regulate a wide variety of physiological processes. Consequently, simple and convenient detection systems for ligands that regulate the function of GPCR have attracted attention as powerful tools for new drug development. We previously developed a yeast-based fluorescence reporter ligand detection system using flow cytometry. However, using this conventional detection system, fluorescence from a cell expressing GFP and responding to a ligand is weak, making detection of these cells by fluorescence microscopy difficult. We here report improvements to the conventional yeast fluorescence reporter assay system resulting in the development of a new highly-sensitive fluorescence reporter assay system with extremely bright fluorescence and high signal-to-noise (S/N) ratio. This new system allowed the easy detection of GPCR signaling in yeast using fluorescence microscopy. Somatostatin receptor and neurotensin receptor (implicated in Alzheimer’s disease and Parkinson’s disease, respectively) were chosen as human GPCR(s). The facile detection of binding to these receptors by cognate peptide ligands was demonstrated. In addition, we established a highly sensitive ligand detection system using yeast cell surface display technology that is applicable to peptide screening, and demonstrate that the display of various peptide analogs of neurotensin can activate signaling through the neurotensin receptor in yeast cells. Our system could be useful for identifying lead peptides with agonistic activity towards targeted human GPCR(s).

## Introduction

G-protein-coupled receptors (GPCRs) represent the largest family of transmembrane receptors and regulate a number of signaling events [1,2]. In humans, these receptors are activated by a large variety of stimuli ranging from small molecules to larger hormones. The stimulation of GPCRs has been reported to engage a broad range of physiological responses, such as blood pressure regulation, smooth muscle contraction, neurotransmission, and cell proliferation [3]. Their key roles in cell signaling have made GPCRs frequent pharmaceutical and therapeutic targets for drug discovery [4]. GPCRs are cell surface transmembrane receptors that transduce signals via heterotrimeric guanine nucleotide binding proteins (G-proteins) comprising Gα-, Gβ- and Gγ-subunits in all eukaryotes [5].

The eukaryotic unicellular yeast, *Saccharomyces cerevisiae*, is a typical host cell used to study heterologous GPCRs at the molecular level [6,7]. Compared with mammalian cell lines, *S. cerevisiae* provides a simple and predictive way for studying GPCR signaling because it expresses only one kind of G-protein [8]. A variety of human GPCRs are known to transduce signals in yeast cells through the endogenous yeast Gα-subunit (Gpa1p) or a yeast-human chimeric G-protein [9,10]. Furthermore, *S. cerevisiae* is used for not only fundamental studies of signaling, but also for research into ligand screening and receptor mutagenesis due to its rapid cell growth and amenability to genetic manipulation compared with other eukaryotes [8,11–13]. 

Yeast cell-surface display technology is a powerful platform that enables proteins expressed in yeast to be tethered onto the cell surface [14–18]. This is accomplished by the use of “anchor” proteins that naturally localize on the cell surface in yeast cells. Typically, the gene encoding the target protein is fused to the anchor protein together with a secretion signal sequence at the N-terminus. This enables both the secretion of the fusion protein and tethers the protein firmly to the cell surface. Two typical anchor proteins are the C-terminal domains of truncated α-agglutinin (Sag1p; a manno-protein involved in sexual adhesion), and truncated Flo1p (a lectin-like cell-wall protein involved in flocculation) containing the glycosyl-phosphatidylinositol (GPI) anchor attachment signal sequence. Both proteins are used to fuse the target protein at their N-terminus [19,20]. To date, yeast cell-surface display technology has been adopted for a broad range of applications including enzymatic catalysis, immune adsorption, and protein engineering [19-22].

Yeast cell-surface display of low molecular peptides on the cell surface forms the basis of several ligand assay systems [23,24]. Expressing a peptide ligand fused to an anchor protein together with a cognate GPCR in a single yeast cell can lead to a series of biological processes within the cell that include expression of peptide ligands, binding to a receptor, signal transmission, and trapping of peptides on the cell wall [23]. In principle, because the tethered peptides are unable to diffuse out of the expression cell, they do not interfere or interact with neighboring cells. This technique forms the basis of the Cell Wall Trapping of Autocrine Peptides (CWTrAP) system. In CWTrAP, transformation of a plasmid library allows the display of various peptides in the engineered yeast with a signal-responsive reporter gene. This allows the facile production of a yeast cell library expressing different candidate peptides and the concurrent screening of target peptides.

Three reporter genes, *HIS3* (auxotrophy), *lacZ* (colorimetry), and *luc* (luminometry), are often used to detect the activation of GPCR signaling in yeast cells [8,11,25]. These reporter genes offer comprehensive screening, comparative quantification, and extremely high detection sensitivity, respectively. In contrast, the green fluorescent protein (GFP) reporter gene provides the most simple procedure for the preparation of measurement samples, by merely washing the cells [6,7,9,10,23]. The most common detection methods for GFP are fluorescence microscopy and flow cytometry (FCM). The detection sensitivity and throughput of FCM are high, enabling comparative quantification [26,27], population analysis [28], and quantitative cell sorting [29,30]. However, not all researchers have access to expensive FCM equipment.

On the other hand, fluorescence microscopes are comparatively common and inexpensive, are simple to operate, and allow cell visualization. However, unlike other reporter gene assay systems, fluorescence microscopy cannot amplify the fluorescence signal by prolonging the reaction time between the enzyme and the substrate [31]. This make it difficult to capture fluorescence images of cells responding to the ligand using conventional yeast GPCR assay systems, even when enhanced green fluorescent protein (EGFP) reporter gene is expressed. There is therefore need to improve the fluorescence reporter system in yeast GPCR assays.

We here report a highly-sensitive fluorescence reporter system applicable to both the yeast-based GPCR assay and CWTrAP technology. By using the tetrameric *Zoanthus* sp. green fluorescent protein (*ZsGreen*), extremely high sensitivity (high signal-to-noise (S/N) ratio) and bright fluorescence were attained, allowing easy observation using fluorescence microscopy. First, we employed human somatostatin receptor subtype-5 (hSSTR5), used previously, to demonstrate the dramatic improvement in the sensitivity and brightness by comparing with a conventional yeast-based fluorescence reporter system. The usefulness of this approach was exhibited with yeast cell-surface display technology. Second, we validated the applicability of this methodology using human somatostatin receptor subtype-2 (hSSTR2) and human neurotensin receptor subtype-1 (hNTSR1) and demonstrated sufficient brightness to permit verification of trapped peptide ligands on the yeast cell wall. Third, we demonstrated that the display of various peptidic neurotensin analogs can activate hNTSR1-mediated signaling in yeast cells.

## Results and Discussion

### General strategy

**Figure 1:**
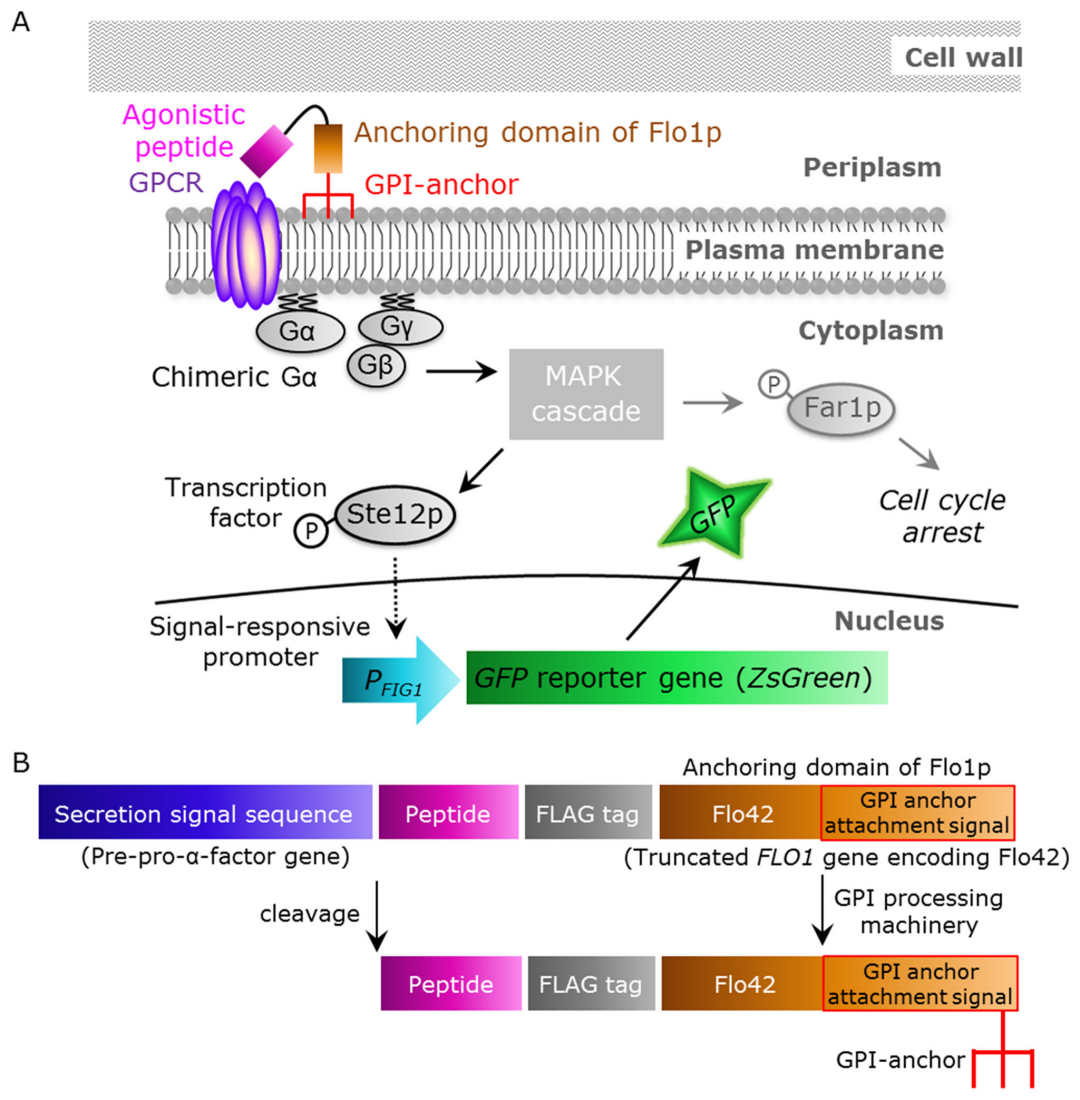
Schematic illustration of signal activation of human GPCRs by membrane-tethered peptide ligands. (*A*) Overview of this study. The membrane-tethered peptide activates human GPCR, which is heterologously produced in yeast, thereby activating the chimeric Gα proteins. This promotes the mitogen-activated protein kinase (MAPK) cascade and transcription factor Ste12p. Phosphorylated Ste12p induces transcription of the GFP reporter gene by binding to a pheromone response element in the *FIG1* promoter. (*B*) Functional domains encoded by the membrane-tethered peptide plasmids. After processing by the secretory pathway, the signal sequence and glycosyl-phosphatidylinositol (GPI) targeting sequence are cleaved and the peptide sequence, which contains a free N-terminus, is tethered on the plasma membrane by GPI covalently linked to the C-terminus.

The aim of this study was to establish a detection method for signals from human GPCRs induced by peptide ligands displayed on the yeast cell surface, thereby permitting observation by fluorescence microscopy. To tether agonistic peptides on the yeast cell membrane, a secretion signal sequence was fused to the N-terminus of the peptide, and a fusion of Flag tag and the C-terminal 42 amino acids of Flo1p (Flo42; anchor protein with GPI anchor attachment signal sequence) was fused to the C-terminus of the peptide [23]. An outline of this strategy is shown in Figure 1A and B. Briefly, the yeast cells synthesize the candidate peptides fused with a secretion signal sequence and an anchoring motif. The membrane-anchored cognate agonistic peptides are capable of binding to the cell surface-expressed GPCRs and of transducing the signal into the cell. The GPI-anchored peptides are then cleaved from the plasma membrane by phosphatidylinositol-specific phospholipase C (PI-PLC) and tethered on the cell wall [15,16]. 

To detect the activation of human GPCRs in yeast, the following gene modifications were implemented. The *sst2*Δ allele is deficient in the yeast principal negative regulator of G-protein signaling (RGS). This deletion therefore results in hypersensitivity for the agonistic stimulus [5,32] and improves sensitivity towards low concentrations of ligand. The *far1*Δ allele is deficient in yeast G1-cyclin-dependent kinase inhibitor; this inhibitor induces G1 cell cycle arrest in response to signaling [5,33]. The *FAR1*-deficient strain can therefore grow and plasmid recovery occurs even in signal-activated states [34]. The *ste2*Δ allele is deficient in the yeast single GPCR, thereby allowing expression of human GPCRs in *STE2*-disrupted strains without competitive receptor expression [5-7,35]. 

To detect signal activation, green fluorescence protein (GFP) reporter was integrated into the yeast genome. The expression of GFP is controlled by the signal responsive *FIG1* promoter. Therefore, stimulation by agonistic peptides results in the generation of a green fluorescence signal [6,7,36]. However, since the signal provided by the conventional detection system integrating an *EGFP* gene as the reporter is too weak to use for fluorescence microscopy, we explored a brighter fluorescent protein. 

### Comparison of two green fluorescence proteins (*ZsGreen* and EGFP) as reporter genes

To obtain the bright fluorescence required for easy observation by microscopy, we explored a recently described tetrameric *Zoanthus* sp. green fluorescent protein (ZsGreen) (brightness, 39,130 (quantum yield, 0.91; molar extinction coefficient, 43,000); excitation maximum, 493 nm; emission maximum, 505 nm) [37] as an alternative to EGFP (brightness, 16,100 (quantum yield, 0.70; molar extinction coefficient, 23,000); excitation maximum, 484 nm; emission maximum, 510 nm). 

The performance (transcription, translation, folding and stability) of *ZsGreen* as a reporter gene in yeast was tested by comparing the fluorescence of yeast cells expressing either ZsGreen or EGFP using fluorescence microscopy and flow cytometry. Single-copy autonomous replicating plasmids, pGK416-ZsGreen and pGK416-EGFP, were constructed to express each green fluorescent protein constitutively under the control of the *PGK1* promoter. Yeast BY4741 wild-type strains harboring these plasmids or mock vector were generated and their green fluorescence was evaluated.

**Figure 2:**
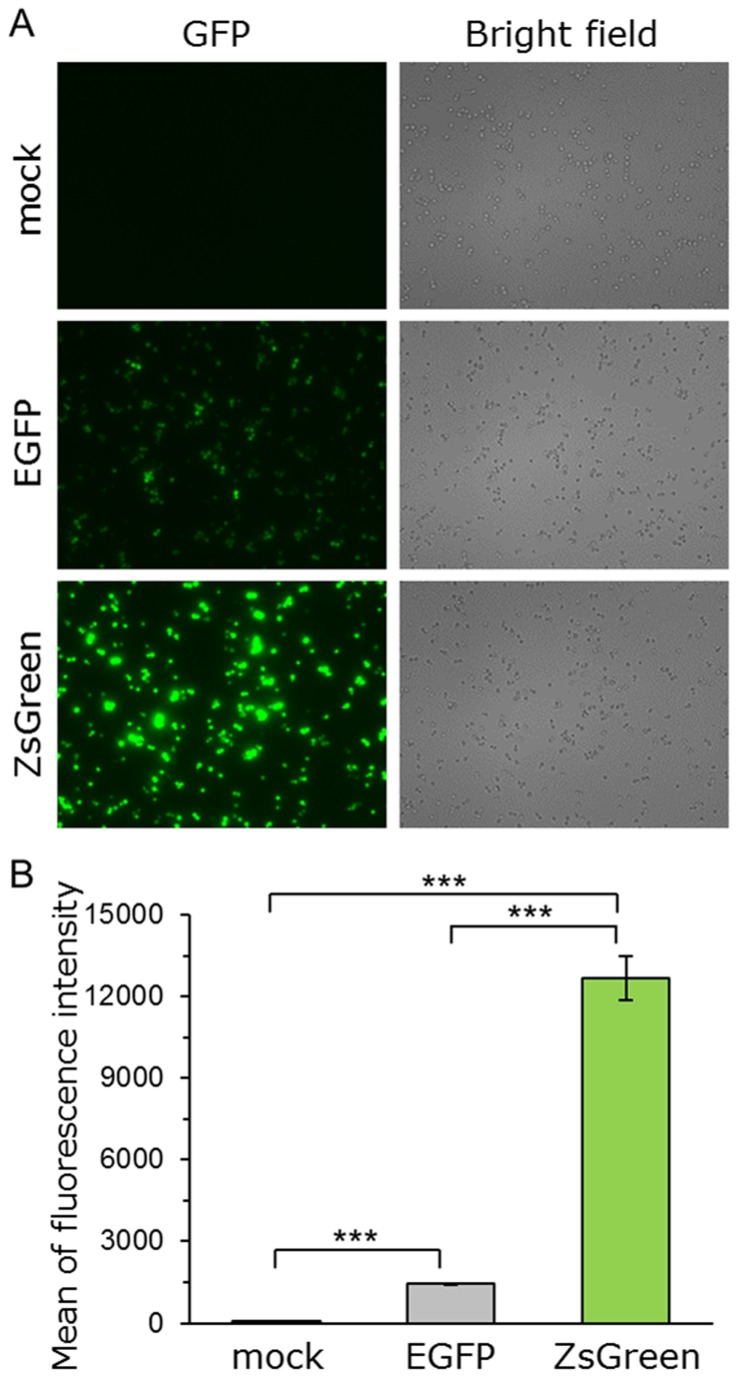
Comparison of green fluorescence proteins (ZsGreen and EGFP). Yeast strain BY4741 was transformed with pGK416-ZsGreen, pGK416-EGFP or pGK416 (empty vector). All transformants were grown in SD selective medium for 18 h. (*A*) Visualization of the green fluorescence (ZsGreen and EGFP). Cells were examined using the 40× objective lens of a fluorescence microscope. Exposure time was 1 s. The left photographs are fluorescence micrographs, and the right photographs are bright-field micrographs. (*B*) The mean GFP fluorescence of 10,000 cells was measured by flow cytometry. Error bars represent the standard deviation from three separate runs (*n* = 3); ***, *p* < 0.001, by one-way ANOVA, Tukey’s post test.

Using fluorescence microscopy, the cells expressing ZsGreen showed clearly brighter fluorescence than those expressing EGFP (Figure 2A). To compare the fluorescence intensity of these cells quantitatively, the average green fluorescence intensity per cell was measured by flow cytometry. The cells expressing ZsGreen exhibited a greater than 8.6-fold increase in fluorescence intensity compared with those expressing EGFP (Figure 2B). This is likely due to better protein features of ZsGreen than EGFP, including expression, translation and fluorescence property. In other cells (medaka melanoma cells, Chinese hamster ovary (CHO) cell line), the cells expressing ZsGreen also emitted brighter green fluorescence than those expressing EGFP, although there was a difference in GFP expression vector (promoter) [38,39]. Taken together, these results indicated that ZsGreen performs better than EGFP as a reporter in yeast.

### Demonstration of improved signal activation detection using human somatostatin receptor subtype-5 (hSSTR5)

Somatostatin (SST), a cyclic neuropeptide which is a growth hormone release-inhibiting factor, is a natural ligand of somatostatin receptors. Five subtypes of somatostatin receptors have been identified (SSTR1–SSTR5) [40,41]. These receptors are important therapeutic targets for acromegaly, Cushing’s syndrome and Alzheimer’s disease [42–44]. 

The human SSTR5 receptor is known to transduce signals in yeast cells through the endogenous yeast Gα-subunit (Gpa1p). A yeast-human chimeric G-protein, in which the carboxyl-terminal 5 amino acid residues of Gpa1p (KIGII) are replaced with the equivalent residues from human Gα_i3_ (ECGLY) (Gpa1/Gα_i3_ transplant, G_i3_tp), has been shown to improve signal transmission via Gα_i_-specific SSTR5 in yeast [6,31,45].

**Figure 3:**
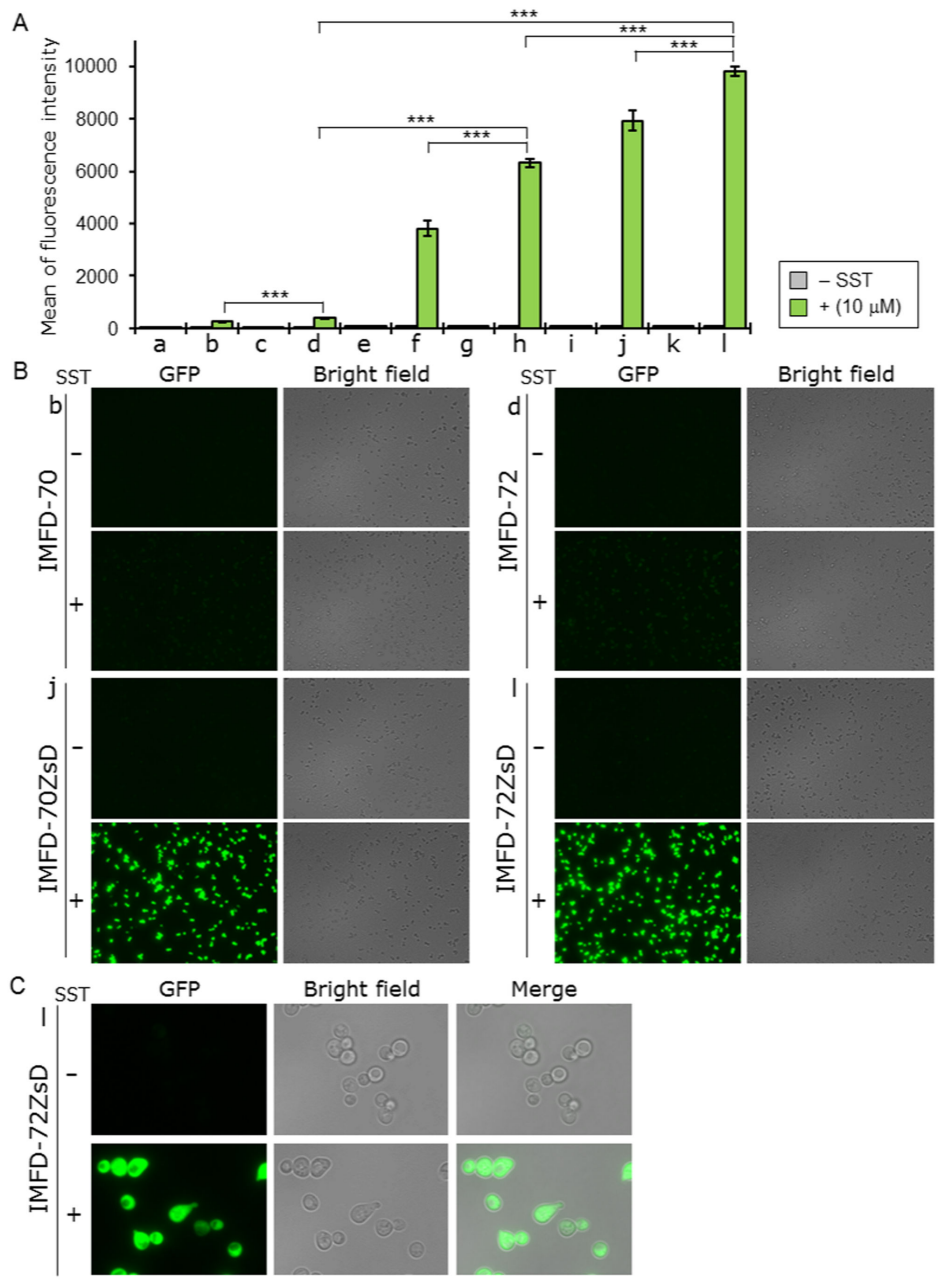
Activation of human somatostatin receptor subtype-5 (hSSTR5) produced in yeast by exogenously-added somatostatin. Yeast strains IMFD-70 (a, b); IMFD-72 (c, d); IMFD-70Zs (e, f); IMFD-72Zs (g, h); IMFD-70ZsD (i, j) and IMFD-72ZsD (k, l) were transformed with either pGK421 (empty vector) (a, c, e, g, i, k) or pGK421-SSTR5 (b, d, f, h, j, l). All transformants were grown in SD selective medium for 18 h. The cells then were incubated for another 4 h in pH-adjusted SD selective medium with or without 10 μM somatostatin (SST, 14 aa peptide). (*A*) The GFP fluorescence of 10,000 cells was measured by flow cytometry. Mean values of the green fluorescence signal of 10,000 cells. Error bars represent the standard deviation from three separate runs (*n* = 3); ***, *p* < 0.001, by one-way ANOVA, Tukey’s post test. (*B*, *C*) Visualization of the green fluorescence. (*B*) Cells were examined using the 40× objective lens of a fluorescence microscope. Exposure time was 1 s. The left photographs are fluorescence micrographs, and the right photographs are bright-field micrographs. (*C*) Cells were examined using the 100× objective lens of a fluorescence microscope. Exposure time was 0.2 s.

The IMFD-70 (Gpa1p) and IMFD-72 (G_i3_tp) strains were selected as the parental yeast strains as they contain two sets of *P*_*FIG1*_ *-EGFP-T*_*FIG1*_ cassettes (*P*_*FIG1*_, *FIG1* promoter; *T*_*FIG1*_, *FIG1* terminator) integrated into the genome (Table 1). One or both sets of *P*_*FIG1*_ *-EGFP-T*_*FIG1*_ cassettes were replaced with *P*_*FIG1*_ *-ZsGreen-T*_*FIG1*_ cassettes to generate IMFD-70Zs and IMFD-72Zs (single substitutions), and IMFD-70ZsD and IMFD-72ZsD (double substitutions) (Table 1). To evaluate the activity of *ZsGreen* as a reporter gene for detecting the signal from human GPCRs, we quantified the induction of green fluorescence responding to exogenously-added SST binding in hSSTR5-expressing yeast cells. Yeast IMFD-70, IMFD-72, IMFD-70Zs, IMFD-72Zs, IMFD-70ZsD and IMFD-72ZsD strains harboring pGK421-SSTR5 or mock vector were generated and examined using a signaling assay. As shown in Figure 3A, all yeast cells harboring the mock vector showed no fluorescence. In the case of yeast cells harboring pGK421-SSTR5, the addition of agonist to yeast strains IMFD-70 and IMFD-72 induced 3.9- and 5.6-fold higher fluorescence; however, the fluorescence was too weak to allow observation with a fluorescence microscope (Figure 3A and B). In contrast, strains IMFD-70Zs and IMFD-72Zs displayed a 33- and 65-fold increase, and strains IMFD-70ZsD and IMFD-72ZsD displayed a 83- and 104-fold increase in fluorescence intensity in response to agonist stimulation (Figure 3A). Using fluorescence microscopy, both the hSSTR5-expressing IMFD-70ZsD and IMFD-72ZsD exhibited changes in green fluorescence and morphology [46] in response to the SST-induced signal (Figure 3B and C). 

**Table 1:** Yeast strains and plasmids used in this study.

Strain or plasmid	Description	Reference
***Strain***		
BY4741	*MAT**a** his3*Δ*1 leu2*Δ*0 met15*Δ*0 ura3*Δ*0*	[63]
IMFD-70	BY4741 *sst2*Δ::*AUR1-C ste2*Δ::*LEU2 fig1Δ*::*EGFP his3*Δ::*P*_*FIG1*_ *-EGFP far1*Δ	[7]
IMFD-72	IMFD-70 *gpa1*Δ::*Gi3tp*	[9]
IMFD-70Zs	BY4741 *sst2*Δ::*AUR1-C ste2*Δ::*LEU2 fig1Δ*::*EGFP his3*Δ::*P*_*FIG1*_ *-ZsGreen far1*Δ	This study
IMFD-72Zs	IMFD-70Zs *gpa1*Δ::*Gi3tp*	This study
IMFD-70ZsD	BY4741 *sst2*Δ::*AUR1-C ste2*Δ::*LEU2 fig1Δ*::*ZsGreen his3*Δ::*P*_*FIG1*_ *-ZsGreen far1*Δ	This study
IMFD-72ZsD	IMFD-70ZsD *gpa1*Δ::*Gi3tp*	This study
***Plasmid***		
pGK416	Yeast expression vector containing *PGK1* promoter, *CEN/ARS* origin and *URA3* marker	[62]
pGK416-ZsGreen	*ZsGreen* in pGK416	This study
pGK416-EGFP	*EGFP* in pGK416	[62]
pBlueScript II KS(+)	Cloning vector	Agilent Technologies
pBlue-FIG1p-URA3	*P*_*FIG1*_ *(*300 bp)-URA3 in pBlueScript II KS(+)	This study
pBlue-FIG1pt-ZsGreen	*P*_*FIG1*_(300 bp)*-URA3-ZsGreen-T*_*FIG1*_(200 bp) in pBlue-FIG1p-URA3	This study
pBlue-HIS3t-URA3	*T*_*HIS3*_ *(*300 bp)-URA3 in pBlueScript II KS(+)	This study
pBlue-HIS3t-FIG1p-ZsGreen	*T*_*HIS3*_(300 bp)*-URA3-P*_*FIG1*_(450 bp)*-ZsGreen* in pBlue-HIS3t-URA3	This study
pBlue-HIS3pt-FIG1pt-ZsGreen	*T*_*HIS3*_(300 bp)*-URA3-P*_*FIG1*_(450 bp)*-ZsGreen-T*_*FIG1*_(300 bp)*-P*_*HIS3*_(200 bp) in pBlue-HIS3t-FIG1p-ZsGreen	This study
pGK421	Yeast expression vector containing *PGK1* promoter, *2μ* origin and *MET15* marker	[6,7]
pGK421-SSTR5	*hSSTR5* in pGK421	[9]
pGK421-SSTR2	*hSSTR2* in pGK421	[31]
pGK421-NTSR1	*hNTSR1* in pGK421	[31]
pGK426-S1442	*SST–Flag–Flo42* in pGK426	[23]
pGK426-alpha42	α*-factor–Flag–Flo42* in pGK426	[23]
pGK426-NTS42	*NTS–Flag–Flo42* in pGK426	This study
pGK426-NTS(8-13)42	*NTS*_*8-13*_ *-Flag–Flo42* in pGK426	This study
pGK426-NMN42	*NMN–Flag–Flo42* in pGK426	This study

**Figure 4:**
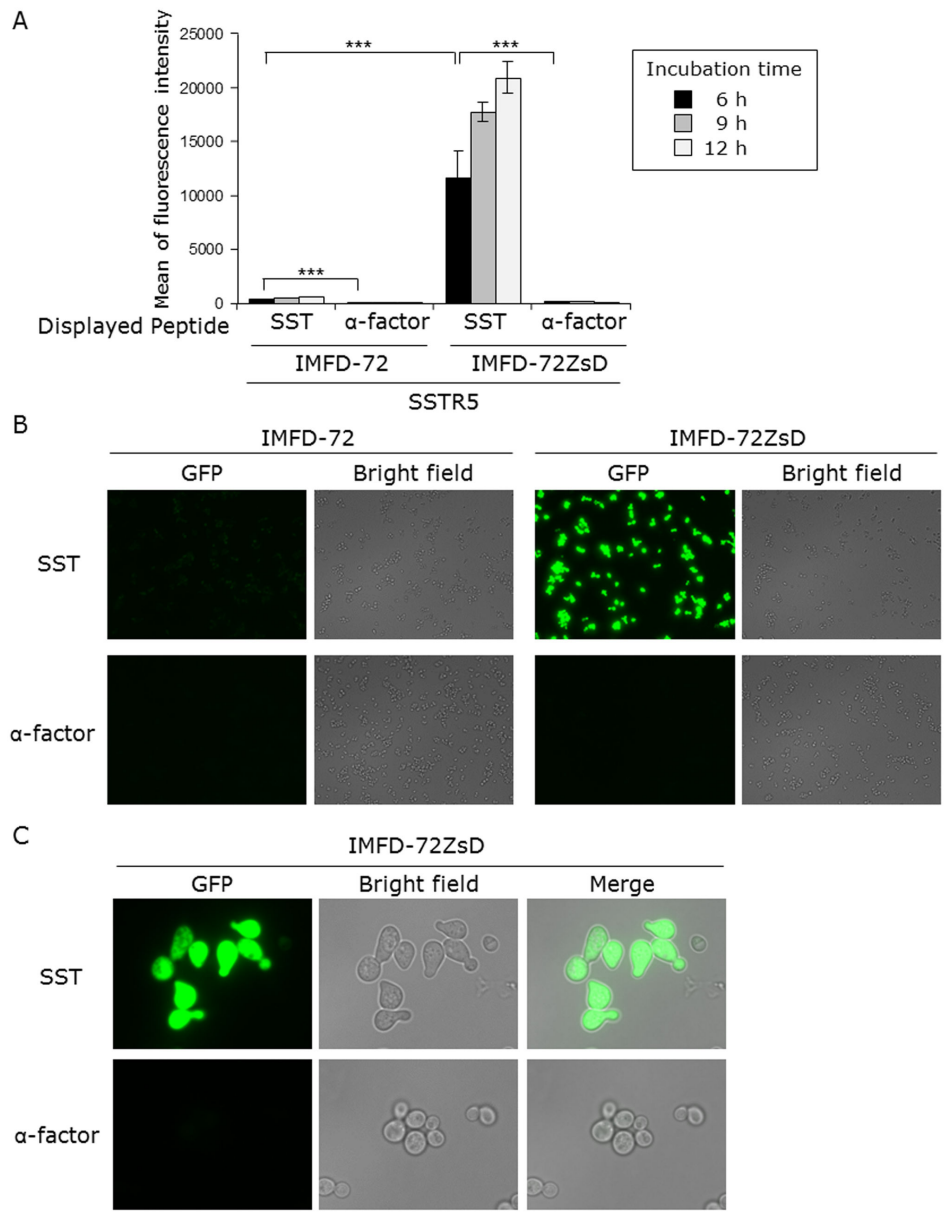
Activation of human somatostatin receptor subtype-5 (hSSTR5) by membrane-tethered somatostatin. Yeast strains IMFD-72 and IMFD-72ZsD, which coexpress pGK421-SSTR5 and either pGK426-S1442 (SST) or pGK426-alpha42 (α-factor), were incubated in pH-adjusted SD selective medium. (*A*) The GFP fluorescence of 10,000 cells was measured by flow cytometry. Mean values of the green fluorescence signal of 10,000 cells. Error bars represent the standard deviations (*n* = 3); ***, *p* < 0.001, by one-way ANOVA, Tukey’s post test. (*B*, *C*) Fluorescence microscopy analysis of cells incubated for 9 h. (*B*) Cells were examined using the 40× objective lens of a fluorescence microscope. Exposure time was 0.67 s. The left photographs are fluorescence micrographs, and the right photographs are bright-field micrographs. (*C*) Cells were examined using the 100× objective lens of a fluorescence microscope. Exposure time was 0.14 s.

Next, we examined whether this system is applicable to yeast cell-surface display technology in addition to observation by fluorescence microscopy. The hSSTR5 expression plasmid (pGK421-SSTR5) and peptide expression plasmids (pGK426-S1442 or pGK426-alpha42) were co-expressed in IMFD-72 and IMFD-72ZsD strains (Table 1). As shown in Figure 4A, the control strains harboring hSSTR5 and the yeast Ste2p receptor agonist, α-factor (α-factor–Flag–Flo42) as the control peptide, did not induce *GFP* reporter gene expression. In contrast, the yeast strains concomitantly expressing hSSTR5 and SST–Flag–Flo42 induced both *EGFP* and *ZsGreen* reporter gene expression. However, since EGFP fluorescence in IMFD-72 was weak, fluorescence microscopy could not distinctly differentiate between the strains displaying control (α-factor) and target (SST) peptides (Figure 4B), whereas IMFD-72ZsD strain using ZsGreen as a reporter clearly showed brighter green fluorescence and morphology change in response to the SST-induced signal (Figure 4B and C). These results indicated that our system provides dramatically improved sensitivity and brightness compared with a conventional yeast-based fluorescence reporter system.

### Activation of human somatostatin receptor subtype-2 (hSSTR2)

**Figure 5:**
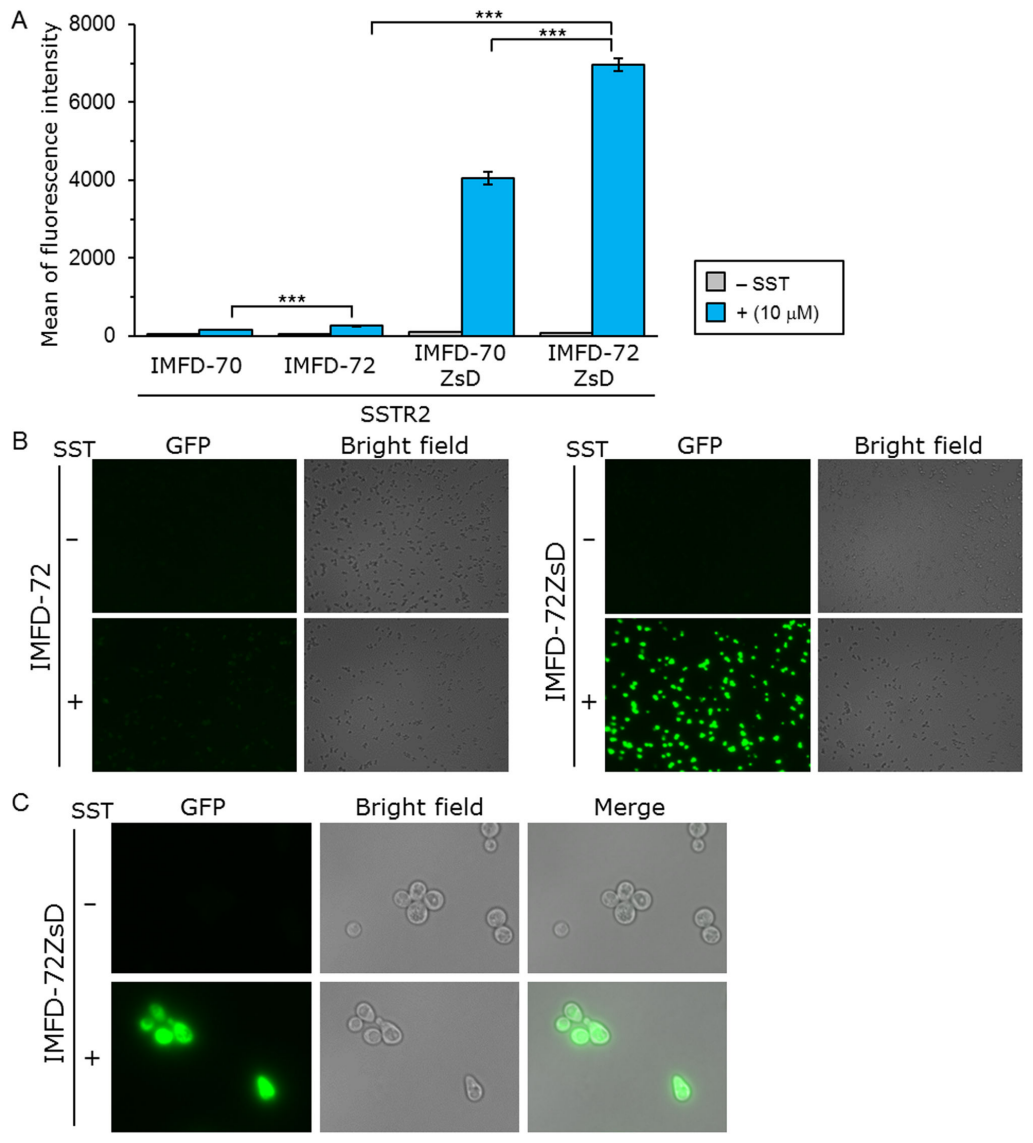
Activation of human somatostatin receptor subtype-2 (hSSTR2) by exogenously-added somatostatin. Yeast strains IMFD-70, IMFD-72, IMFD-70ZsD and IMFD-72ZsD were transformed with pGK421-SSTR2. All transformants were grown in SD selective medium for 18 h. The cells then were incubated for another 4 h in pH-adjusted SD selective medium with or without 10 μM somatostatin (SST, 14 aa peptide). (*A*) The GFP fluorescence of 10,000 cells was measured by flow cytometry. Mean values of the green fluorescence signal of 10,000 cells. Error bars represent the standard deviations (*n* = 3); ***, *p* < 0.001, by one-way ANOVA, Tukey’s post test. (*B*, *C*) Visualization of the green fluorescence. (*B*) Cells were examined using the 40× objective lens of a fluorescence microscope. Exposure time was 1 s. The left photographs are fluorescence micrographs, and the right photographs are bright-field micrographs. (*C*) Cells were examined using the 100× objective lens of a fluorescence microscope. Exposure time was 0.33 s.

To validate the applicability of this system for human somatostatin receptor subtype-2 (hSSTR2), the activation of hSSTR2 by exogenously-added SST was quantified. Yeast IMFD-70, IMFD-72, IMFD-70ZsD and IMFD-72ZsD strains harboring pGK421-SSTR2 were generated and studied using the signaling assay. As shown in Figure 5A, whereas the addition of agonist to yeast strains IMFD-70 and IMFD-72 induced a 3.0- and 4.6-fold higher fluorescence, agonist stimulation of strains IMFD-70ZsD and IMFD-72ZsD induced a 36- and 81-fold drastic increase in the fluorescence intensity. Using fluorescence microscopy, the hSSTR2-expressing IMFD-72ZsD clearly exhibited changes in green fluorescence and morphology in response to the SST-induced signal (Figure 5B and C). 

**Figure 6:**
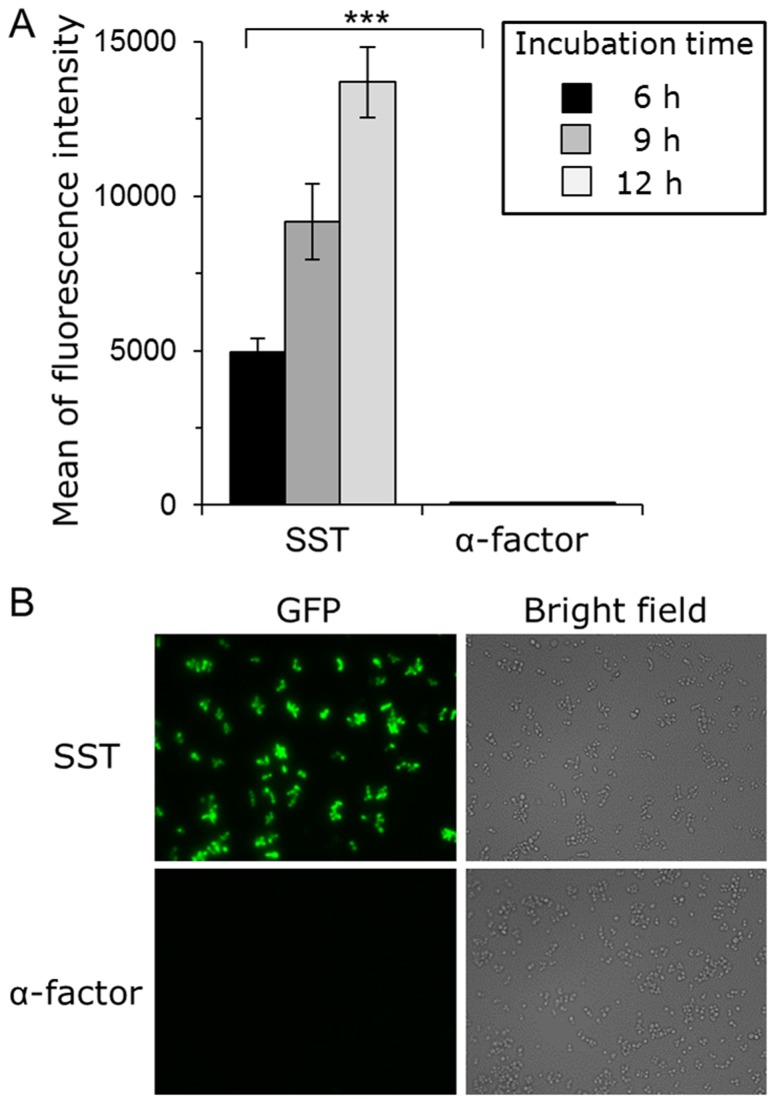
Activation of human somatostatin receptor subtype-2 (hSSTR2) by membrane-tethered somatostatin. Yeast strain IMFD-72ZsD which coexpresses pGK421-SSTR2 and either pGK426-S1442 (SST) or pGK426-alpha42 (α-factor) was incubated in pH-adjusted SD selective medium. (*A*) The GFP fluorescence of 10,000 cells was measured by flow cytometry. Mean values of the green fluorescence signal of 10,000 cells. Error bars represent the standard deviations (*n* = 3); ***, *p* < 0.001, by one-way ANOVA, Tukey’s post test. (*B*) Fluorescence microscopy analysis of the cells incubated for 9 h. Cells were examined using the 40× objective lens of a fluorescence microscope. Exposure time was 0.25 s. The left photographs are fluorescence micrographs, and the right photographs are bright-field micrographs.

Next, to investigate the activation of hSSTR2 by membrane-tethered somatostatin, an hSSTR2 expression plasmid (pGK421-SSTR2) and peptide expression plasmids (pGK426-S1442 or pGK426-alpha42) were co-expressed in the IMFD-72ZsD strain (Table 1). As expected, the average fluorescence of the yeast strain concomitantly expressing hSSTR2 and SST–Flag–Flo42 increased with increasing incubation time, exhibiting a greater than 169-fold increase in fluorescence intensity compared with the yeast strain possessing hSSTR2 and α-factor–Flag–Flo42 after 12 h cultivation (Figure 6A). Using a fluorescence microscope, the yeast strain concomitantly expressing hSSTR2 and SST–Flag–Flo42 clearly showed a brighter green fluorescence change in response to the SST-induced signal (Figure 6B). These results indicated that our system is applicable to use with yeast cell-surface display technology.

### Activation of human neurotensin receptor subtype-1 (hNTSR1)

Neurotensin (NTS) is a 13-amino-acid peptide found in the nervous system and in peripheral tissues [47,48]. NTS shows a wide range of biological activities and has important roles in Parkinson’s disease and the pathogenesis of schizophrenia, the modulation of dopamine neurotransmission, hypothermia, antinociception, and in promoting the growth of cancer cells [49–53]. Three neurotensin receptors have been identified. NTSR1 [54] and NTSR2 [55] belong to the class A GPCR family, whereas NTSR3 (also called SORT1) is a member of the sortilin family and contains a single transmembrane domain [56]. The crystal structure of the complex of NTSR1 and a partial NTS peptide was recently solved [48]. Although many structures of the complex of GPCR and agonist or antagonist had been reported, this was the first report of the structure of NTSR1 bound with peptide hormone [48]. Thus, attention was attracted to the study of NTSR1-mediated signaling in order to understand the GPCR-ligand binding modes.

**Figure 7:**
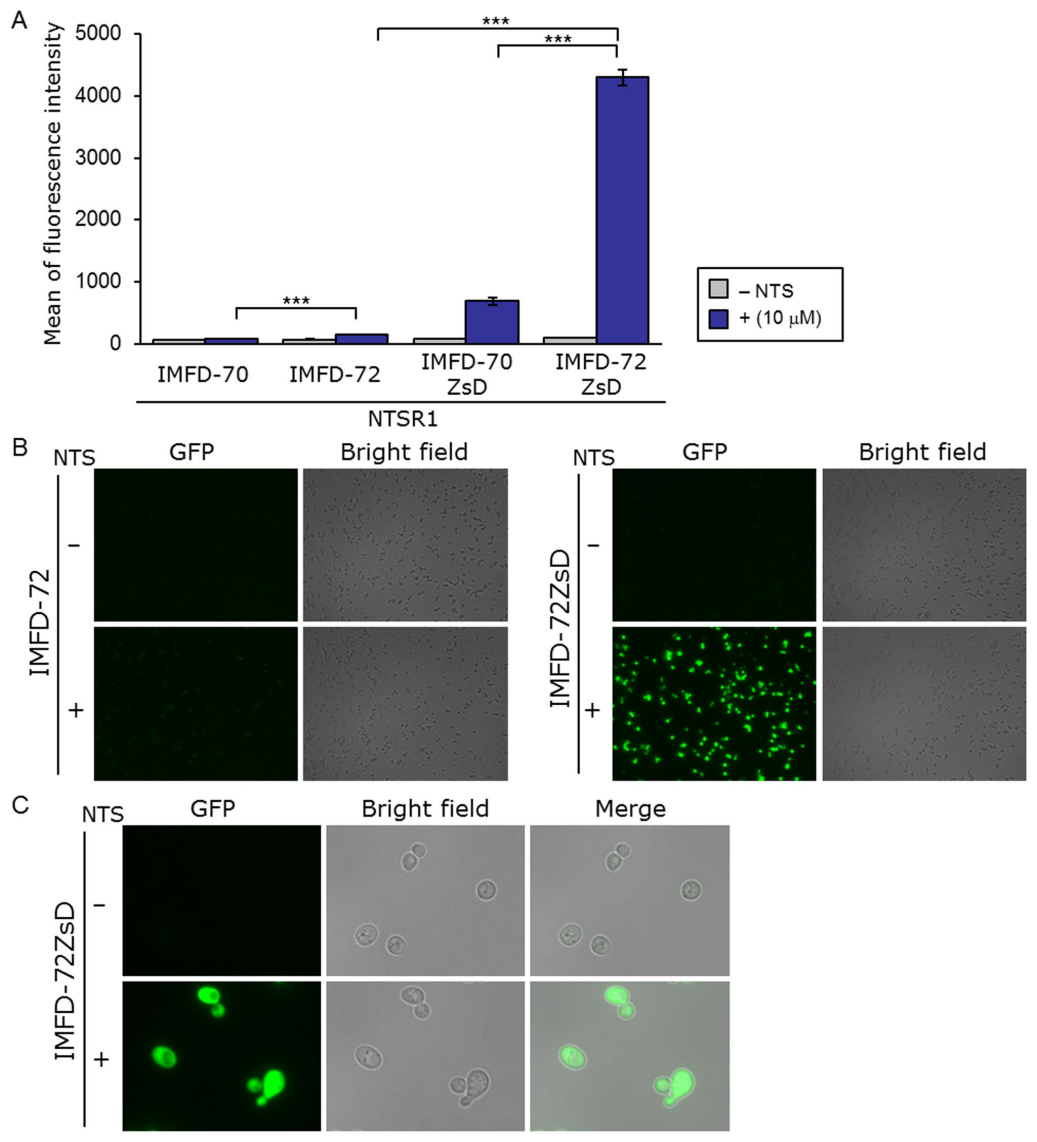
Activation of human neurotensin receptor subtype-1 (hNTSR1) produced in yeast following exogenously-added neurotensin. Yeast strains IMFD-70, IMFD-72, IMFD-70ZsD and IMFD-72ZsD were transformed with pGK421-NTSR1. All transformants were grown in SD selective medium for 18 h. The cells then were incubated for another 4 h in pH-adjusted SD selective medium with or without 10 μM neurotensin (NTS, 13 aa peptide). (*A*) The GFP fluorescence of 10,000 cells was measured by flow cytometry. Mean values of the green fluorescence signal of 10,000 cells. Error bars represent the standard deviation from three separate runs (*n* = 3); ***, *p* < 0.001, by one-way ANOVA, Tukey’s post test. (*B*, *C*) Visualization of the green fluorescence. (*B*) Cells were examined using the 40× objective lens of a fluorescence microscope. Exposure time was 1 s. The left photographs are fluorescence micrographs, and the right photographs are bright-field micrographs. (*C*) Cells were examined using the 100× objective lens of a fluorescence microscope. Exposure time was 0.5 s.

Yeast IMFD-70, IMFD-72, IMFD-70ZsD and IMFD-72ZsD strains harboring pGK421-NTSR1 were generated for study using the signaling assay. As shown in Figure 7A, addition of agonist to yeast strains IMFD-70 and IMFD-72 resulted in only 1.3- and 2.3-fold higher fluorescence than in the absence of agonist, whereas strains IMFD-70ZsD and IMFD-72ZsD displayed a 7.9- and 45-fold increase in fluorescence intensity in response to agonist stimulation. Using fluorescence microscopy, the hNTSR1-expressing IMFD-72ZsD clearly exhibited changes in green fluorescence and morphology in response to the NTS-induced signal (Figure 7B and C). Similar to the results with hSSTR2, the IMFD-72ZsD strain harboring pGK421-NTSR1 exhibited a much higher average GFP intensity (S/N ratio) following stimulation by NTS compared with the IMFD-70ZsD strain harboring pGK421-NTSR1 (Figure 7A). These results indicated that the yeast-human chimeric G-protein subunit (G_i3_tp) significantly improved hNTSR1-mediated signal transmission in yeast cells [31].

**Figure 8:**
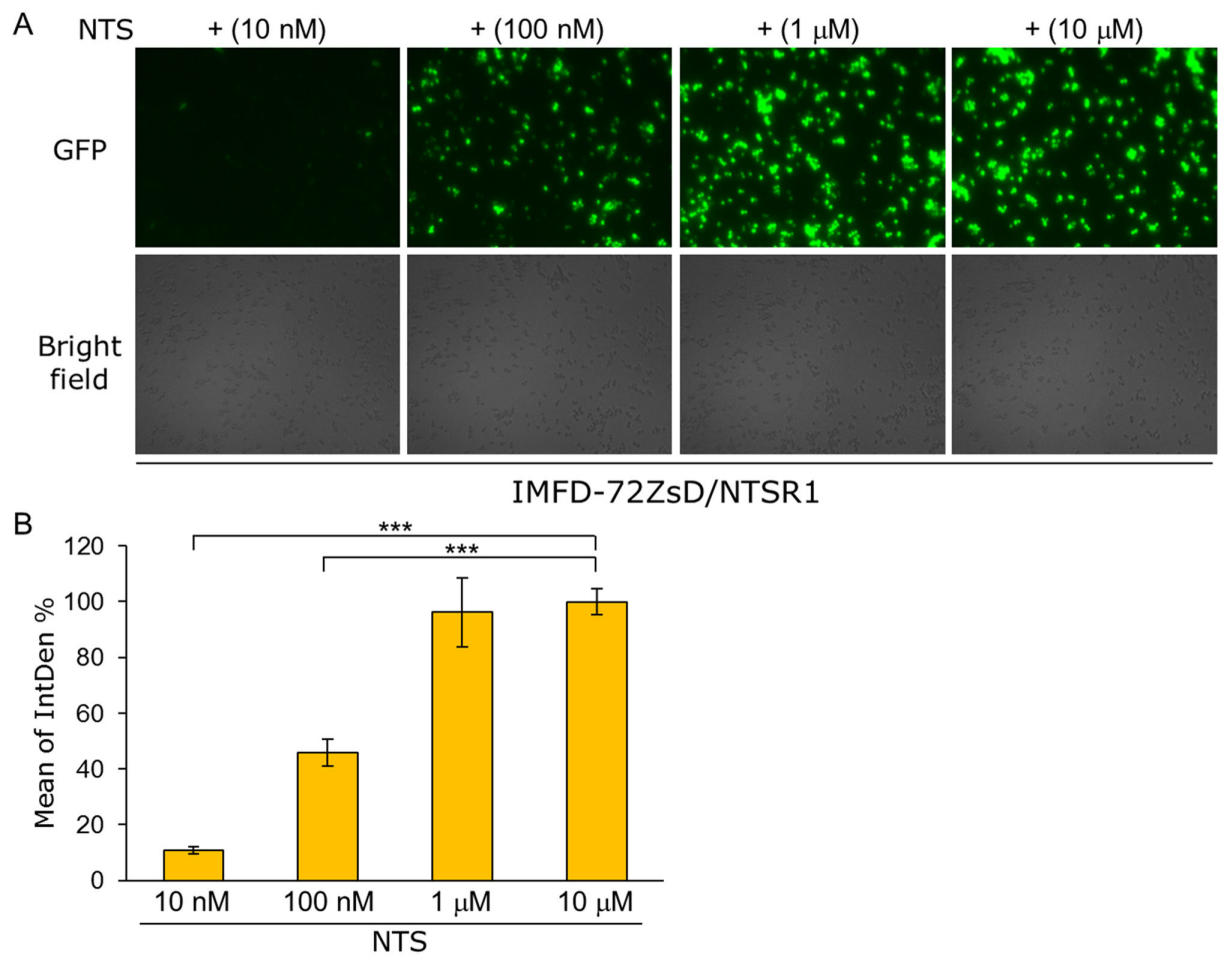
Quantitative digital image analysis of ZsGreen fluorescence using fluorescence microscopy and ImageJ software. Yeast strain IMFD-72ZsD was transformed with pGK421-NTSR1. The transformant was grown in SD selective medium for 18 h. The cells then were incubated for another 4 h in pH-adjusted SD selective medium with neurotensin (NTS, 13 aa peptide) at several concentrations. (*A*) Visualization of the green fluorescence. Cells were examined using the 40× objective lens of a fluorescence microscope. Exposure time was 1 s. The upper photographs are fluorescence micrographs, and the lower photographs are bright-field micrographs. (*B*) The GFP fluorescence of 100 cells was measured as integrated density (IntDen, ImageJ software). Mean values of the green fluorescence signal of 100 cells. IntDen % was represented by relative IntDen normalized with the values of maximal effects of NTS-specific dose-responses. Error bars represent the standard deviation from four separate runs (*n* = 4); ***, *p* < 0.001, by one-way ANOVA, Tukey’s post test.

To test whether the fluorescence microscopy approach is applicable for quantitative analysis, the yeast strain IMFD-72ZsD harboring pGK421-NTSR1 was used for observing green fluorescence images responding to several NTS concentrations. As shown in Figure 8A, the cells displayed the several distinct orders of brightness in ZsGreen fluorescence. Then, using the green fluorescence images, digital image analysis with ImageJ was conducted. The digital intensities of green fluorescence varied in a NTS-dependent manner, indicating that the digital image analysis with fluorescence microscopy is available for the quantitative comparison of the signaling levels (Figure 8B).

### Activation of hNTSR1 by displaying neurotensin analogue peptides on the yeast cell surface

**Figure 9:**
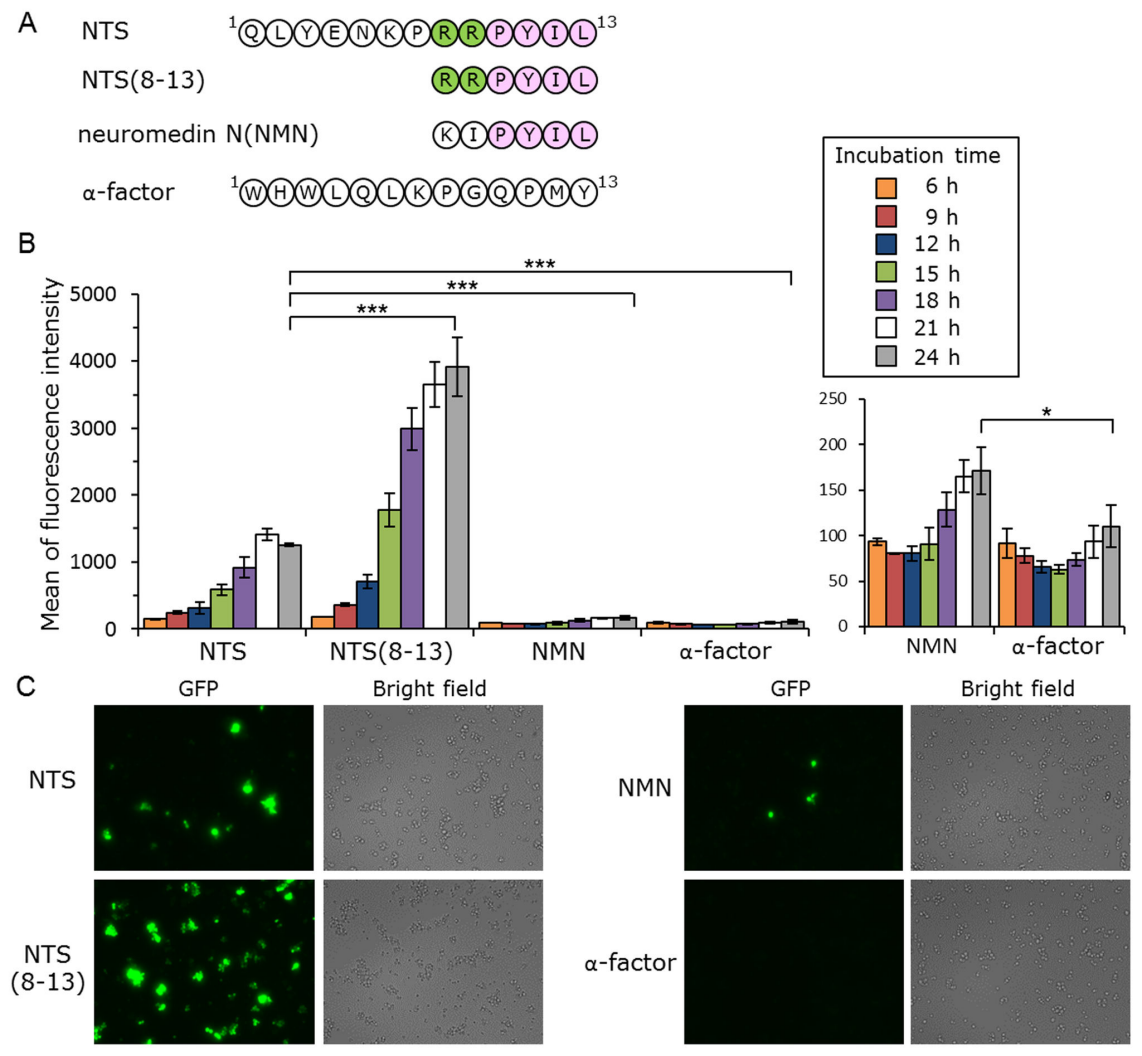
Activation of human neurotensin receptor subtype-1 (hNTSR1) by membrane-tethered neurotensin. (*A*) Amino acid sequences of membrane-tethered peptides. (*B*, *C*) Yeast strain IMFD-72ZsD, which coexpresses pGK421-NTSR1 and either pGK426-NTS42 (NTS), pGK426-NTS(8-13)42 (NTS(8-13)), pGK426-NMN42 (NMN) or pGK426-alpha42 (α-factor), was incubated in pH-adjusted SD selective medium. (*B*) The GFP fluorescence of 10,000 cells was measured by flow cytometry. Mean values of the green fluorescence signal of 10,000 cells. Error bars represent the standard deviations (*n* = 3); *, *p* < 0.05, and ***, *p* < 0.001, by one-way ANOVA, Tukey’s post test. (*C*) Fluorescence microscopy analysis of the cells incubated for 24 h. Cells were examined using the 40× objective lens of a fluorescence microscope. Exposure time was 0.67 s. The left photographs are fluorescence micrographs, and the right photographs are bright-field micrographs.

Finally, several neurotensin sequence analogues were employed to demonstrate that the display of various peptidic neurotensin analogs could also activate hNTSR1-mediated signaling in yeast cells (Figure 9A). Neurotensin-(8-13) (NTS_8–13_), a pharmacophore of neurotensin, has higher potency and efficacy than full-length NTS [48,57]. Studies on the structure-activity relationship of neurotensin verified that positive charges at positions 8 and 9 are important but not prerequisite for its bioactivity [58]. There is charge complementarity between NTS_8–13_ and its binding pocket: the positively-charged arginine side chains of NTS_8–13_ are adjacent to the electronegative rim of the binding site, and the C terminus of NTS_8–13_ is predicted to form a salt bridge with R327 [48]. A neurotensin-like peptide, neuromedin N (NMN), has high homology with the C-terminal sequence of neurotensin and is encoded by the same gene as neurotensin [59,60]. Although the amino acid sequence of NMN (KIPYIL) is similar to that of NTS_8–13_ (RRPYIL) (Figure 9A), NMN shows very weak biological activity [60].

An hNTSR1 expression plasmid (pGK421-NTSR1) and peptide expression plasmids (pGK426-NTS42, pGK426-NTS(8-13)42, pGK426-NMN42 or pGK426-alpha42) were co-expressed in IMFD-72ZsD strain (Table 1). As shown in Figure 9B, the average fluorescence of the yeast strain concomitantly expressing hNTSR1 and NTS–Flag–Flo42 increased with increasing incubation time, exhibiting a greater than 11-fold increase in fluorescence intensity compared with the strain possessing hNTSR1 and α-factor–Flag–Flo42 after 24 h cultivation. The membrane-tethered NTS_8–13_ and NMN also induced ZsGreen fluorescence, exhibiting a greater than 35- and 1.5-fold increase in fluorescence intensity compared with α-factor after 24 h cultivation (NTS_8–13_ > NTS > NMN > α-factor) (Figure 9B). Fluorescence microscopy demonstrated that the observed differences in fluorescence brightness arose from differences in the membrane-tethered neurotensin sequence (Figure 9C) and showed that the display of various peptidic neurotensin analogs can activate hNTSR1-mediated signaling in yeast cells. To confirm the value of this assay, a small scale screening was carried out with membrane-tethered NTS as a positive control and membrane-tethered α-factor as a negative control, respectively (Figure S1). The Z’ factor that reflects both the assay signal dynamic range and the data variation associated with the measured signal [61] was calculated to assess the assay quality. The value yielded upward of 0.5 (Z’ factor = 0.57), showing the robustness and suitability for screening of the assay. It is expected that this technology will be applicable to the identification of peptide ligand pharmacophores, and for screening random or combinatorial peptide libraries for functional activation of GPCRs. The methodology presented in this study could be useful for the discovery of novel peptide ligands of human GPCRs.

In conclusion, we have established a fluorescence microscopy-based microbial yeast biosensor to detect human GPCR signaling that is applicable to CWTrAP technology and exhibits extremely bright fluorescence and high signal-to-noise (S/N) ratio. The biosensor employs a new highly-potent fluorescence reporter (ZsGreen) and Gα-engineered yeast strain. This system permitted the quick and easy visual distinction of cells responding to the agonist using fluorescence microscopy, thereby allowing facile detection of agonistic ligands of human GPCRs. Since our system is applicable to not only hSSTR5 but also hSSTR2 and hNTSR1, this system can likely be extended to other human GPCRs, which comprise one of the most important types of drug targets being pursued today. Application of this method will allow the identification of lead peptides from combinatorial peptide libraries to provide starting points for drug screening.

## Materials and Methods

### Media

Synthetic dextrose (SD) medium contained 6.7 g/L yeast nitrogen base without amino acids (YNB) (BD-Diagnostic Systems, Sparks, MD, USA) and 20 g/L glucose. For SDM71 media, SD medium was adjusted to pH 7.1 with 200 mM MOPSO buffer (Nacalai Tesque, Kyoto, Japan). YPD medium contained 10 g/L yeast extract (Nacalai Tesque), 20 g/L peptone (BD-Diagnostic Systems) and 20 g/L glucose. As appropriate, SD medium was supplemented with amino acids or nucleotides (20 mg/L histidine, 60 mg/L leucine, 20 mg/L methionine, or 20 mg/L uracil). For solid medium, agar was added at 20 g/L.

### Plasmids

All plasmids used in this study are summarized in Table 1. All oligonucleotides used for the plasmid constructions are listed in Table S1. Plasmid maps are presented in Figure S2.

The plasmid used for expression of *Zoanthus* sp. green fluorescent protein (ZsGreen) was constructed as follows. A DNA fragment encoding the *ZsGreen* gene was PCR-amplified from pZsGreen (Takara Bio, Shiga, Japan) using the oligonucleotides o1 and o2, digested with *Nhe*I+*Eco*RI, and inserted into the same sites between the *PGK1* promoter (*P*_*PGK1*_) and the *PGK1* terminator (*T*_*PGK1*_) on pGK416 [62], yielding the plasmid pGK416-ZsGreen.

To express the *ZsGreen* gene under the control of the pheromone-responsive *FIG1* promoter [6,7,36], the *ZsGreen* gene was inserted into a plasmid that integrated into the yeast chromosome at a position upstream of the *FIG1* gene. Plasmid construction was as follows. A DNA fragment containing the homologous sequence at the *FIG1* promoter (upstream of *FIG1* gene; 300 bp) was PCR-amplified from BY4741 [63] genomic DNA using oligonucleotides o3 and o4. A DNA fragment containing the *URA3* selectable marker (along with 40 nucleotides from the 3’ side of the *FIG1* promoter at the 3’ end) was PCR-amplified from pRS426 (American Type Culture Collection, Manassas, VA) using oligonucleotides o5 and o6. The amplified fragments were digested with *Sac*II+*Xba*I and *Xba*I+*Eco*RI (respectively) and ligated together into *Sac*II, *Eco*RI-cleaved pBlueScript II KS(+) vector (Agilent Technologies, Santa Clara, CA, USA). The resultant plasmid was named pBlue-FIG1p-URA3. A DNA fragment containing the *ZsGreen* gene was PCR-amplified from pZsGreen using oligonucleotides o7 and o8. A DNA fragment containing the homologous sequence of the *FIG1* terminator (downstream of *FIG1* gene; 200 bp) was PCR-amplified from BY4741 genomic DNA using oligonucleotides o9 and o10. The amplified fragments were digested with *Eco*RI+*Xho*I and *Xho*I+*Kpn*I (respectively) and ligated together into *Eco*RI, *Kpn*I-cleaved pBlue-FIG1p-URA3. The resultant plasmid was named pBlue-FIG1pt-ZsGreen.

The plasmid used for substituting *P*_*FIG1*_ *-ZsGreen-T*_*FIG1*_ for *P*_*FIG1*_ *-EGFP-T*_*FIG1*_ at the *HIS3* gene locus on the yeast chromosome was constructed as follows. A DNA fragment containing the homologous sequence of the *HIS3* terminator (downstream of *HIS3* gene; 300 bp) was PCR-amplified from BY4741 genomic DNA using oligonucleotides o11 and o12. A DNA fragment containing the *URA3* selectable marker (with 40 nucleotides from the 5’ side of the *HIS3* terminator at the 3’ end) was PCR-amplified from pRS426 (American Type Culture Collection, Manassas, VA) using oligonucleotides o5 and o13. The amplified fragments were digested with *Sac*II+*Xba*I and *Xba*I+*Bam*HI (respectively) and ligated together into *Sac*II, *Bam*HI-cleaved pBlueScript II KS(+) vector (Agilent Technologies, Santa Clara, CA, USA). The resultant plasmid was named pBlue-HIS3t-URA3. A DNA fragment containing the *FIG1* promoter (upstream of *FIG1* gene; 450 bp) was PCR-amplified from BY4741 genomic DNA using oligonucleotides o14 and o15. A DNA fragment containing the *ZsGreen* gene was PCR-amplified from pZsGreen using oligonucleotides o7 and o16. The amplified fragments were digested with *Bam*HI+*Eco*RI and *Eco*RI+*Cla*I (respectively) and ligated together into *Bam*HI, *Cla*I-cleaved pBlue-HIS3t-URA3. The resultant plasmid was named pBlue-HIS3t-FIG1p-ZsGreen. A DNA fragment containing the *FIG1* terminator (downstream of *FIG1* gene; 300 bp) was PCR-amplified from BY4741 genomic DNA using oligonucleotides o17 and o18. A DNA fragment containing the homologous sequence of the *HIS3* promoter (upstream of *HIS3* gene; 200 bp) was PCR-amplified from BY4741 genomic DNA using oligonucleotides o19 and o20. The amplified fragments were digested with *Cla*I+*Sal*I and *Sal*I+*Kpn*I (respectively) and ligated together into *Cla*I, *Sal*I-cleaved pBlue-HIS3t-FIG1p-ZsGreen. The resultant plasmid was named pBlue-HIS3pt-FIG1pt-ZsGreen.

The plasmids used for expression of membrane-tethered peptides, in which the pre, pro α-factor leader region gene (containing secretion signal sequence, s.s.), peptide gene and *FLO42* anchor gene with *FLAG* at the N-terminus are encoded, were constructed as follows. A DNA fragment containing s.s. of α-factor with a partial NTS sequence for overlapping PCR was PCR-amplified from pGK426-S1442 [23] using oligonucleotides o25 and o26. The amplified fragment was then used as the template for overlapping PCR with the oligonucleotides o25 and o27. The s.s.-*NTS* was digested with *Nhe*I+*Sal*I and ligated into similarly digested pGK426-tgFLO42 [23], resulting in the plasmid pGK426-NTS42. A DNA fragment containing s.s. of α-factor and the C-terminal portion of NTS (NTS_8–13_) was PCR-amplified from pGK426-NTS42 using oligonucleotides o25 and o28. The s.s.-*NTS*_*8–13*_ was digested with *Nhe*I+*Sal*I and ligated into similarly digested pGK426-tgFLO42 [23], resulting in the plasmid pGK426-NTS(8-13)42. A DNA fragment containing s.s. of α-factor and neuromedin N (NMN) was PCR-amplified from pGK426-NTS42 using oligonucleotides o25 and o29. The s.s.-*NMN* was digested with *Nhe*I+*Sal*I and ligated into similarly digested pGK426-tgFLO42 [23], resulting in the plasmid pGK426-NMN42.

### Yeast Strains

Yeast strains used in this study were generated from BY4741 [63] as a parental backbone strain and are listed in Table 1. Transformation with linear DNA fragments was performed using the lithium acetate method [64]. To eliminate the *URA3* selectable marker in each transformation step, we followed previous procedures [6,7] modified to incorporate the marker recycling method [65]. 

The strains expressing *Zoanthus* sp. green fluorescence protein (ZsGreen) were generated as follows. The DNA fragment obtained by digesting pBlue-HIS3pt-FIG1pt-ZsGreen with *Sac*II and *Kpn*I was transformed into IMFD-70 [7] and IMFD-72 [9]. After confirming the correct integration, the *URA3* marker was “popped-out” by homologous recombination using counter-selection with 5-fluoroorotic acid (5-FOA, Fluorochem, Derbyshire, UK). The resultant strains were designated as IMFD-70Zs and IMFD-72Zs, respectively. The DNA fragment obtained by digesting pBlue-FIG1pt-ZsGreen with *Sac*II and *Kpn*I was transformed into IMFD-70Zs and IMFD-72Zs. After confirming correct integration, the *URA3* marker was “popped-out” by counter-selection with 5-FOA. The constructed strains were designated as IMFD-70ZsD and IMFD-72ZsD, respectively.

### Fluorescence Microscopy Imaging

The cultured cells were washed and suspended in distilled water. The cell suspensions were observed using a BZ-9000 fluorescence microscope (Keyence, Osaka, Japan). Green fluorescence images were acquired with a 470/40 band-pass filter for excitation and a 535/50 band-pass filter for emission. Digital image intensities of green fluorescence were carried out, from four experiments each, with the ImageJ software (freely downloadable from the ImageJ website, http://rsbweb.nih.gov/ij/) and expressed as Integrated Density (IntDen) or its percent.

### Flow Cytometry Analysis

Flow cytometry measurements of green fluorescence followed previous procedures [6,7]. In brief, GFP was detected using a BD FACSCanto II flow cytometer equipped with a 488-nm blue laser (Becton, Dickinson and Company, Franklin Lakes, NJ, USA); the data were analyzed using BD FACSDiva software (v5.0; Becton, Dickinson and Company). The GFP fluorescence signal was collected through a 530/30 nm band-pass filter and the GFP-A mean of 10,000 cells was defined as ‘green fluorescence intensity’.

### Comparison Assay of Constitutively-expressed Two Green Fluorescence Proteins (ZsGreen and EGFP)

Yeast transformants were grown in SD medium (supplemented as needed) at 30 °C overnight, and the cells then were inoculated into 5 mL of the respective fresh SD medium to give an initial optical density of 0.03 at 600 nm (OD_600_ = 0.03). The cells were incubated at 30 °C on a rotary shaker at 150 rpm for up to 18 h. After incubation, the yeast cells were observed using a fluorescence microscope, and then were diluted with 1 mL of sheath fluid and fluorescence was analyzed by flow cytometry.

### GPCR Signaling Assay with Exogenously Added Ligands

GPCR signaling assays with exogenously added ligands basically followed previous procedures [6,7]. In brief, to assay signal activation from human GPCRs, yeast strains harboring the pGK421-based plasmids were grown in SD medium (supplemented as needed) at 30 °C overnight, then the cells then were inoculated into 5 mL of the respective fresh SD medium to give an initial OD_600_ = 0.03. The cells were incubated at 30 °C on a rotary shaker at 150 rpm for up to 18 h and harvested, washed, and resuspended in water to give an OD_600_ = 10. The suspensions were added (at 10 μL/well; to give an OD_600_ = 1) to the wells of 96-well cluster dishes containing fresh SDM71 medium (80 μL/well) supplemented (10 μL/well) with 10 μM of either somatostatin (SST) or neurotensin (NTS) (Calbiochem, Darmstadt, Germany) or distilled water (no SST (NTS), control). The plates were incubated at 30 °C with shaking (150 rpm) for 4 h, then the yeast cells were observed using a fluorescence microscope, diluted with 1 mL of sheath fluid, and fluorescence was analyzed by flow cytometry.

### GPCR Signaling Assay using Membrane-tethered Peptide Ligands

GPCR signaling assays using membrane-tethered peptide ligands basically followed a previous procedure [23] with some modifications. In brief, to assay signal activation from human GPCRs, yeast strains harboring the pGK421-based GPCR expression plasmids and pGK426-based peptide display plasmids were grown in SD medium (supplemented as needed) at 30 °C overnight, then the cells then were inoculated into 5 mL of the respective fresh SDM71 medium to give an initial OD_600_ = 0.1. The cells were incubated at 30 °C on a rotary shaker at 150 rpm, then the yeast cells were observed using a fluorescence microscope, diluted with 1 ml of sheath fluid, and fluorescence was analyzed by flow cytometry.

### Data Analysis

Data were analyzed using KaleidaGraph4.0. Statistical significance of the differences between more than two groups was calculated by one-way ANOVA, followed by Tukey’s post test. Z’ factor, a commonly used parameter reflecting the robustness and the quality of assays, was determined as follows: Z’ factor = 1 ‒ 3(SD_positive control_ + SD_negative control_)/ (mean_positive control_ ‒ mean_negative control_), where “SD” represents standard deviation of multiple replicates of an assay [61].

## Supporting Information

Figure S1 **Determination of the Z’ factor.** Yeast strain IMFD-72ZsD, which coexpresses pGK421-NTSR1 and either pGK426-NTS42 (NTS, positive control) or pGK426-alpha42 (α-factor, negative control), was incubated in pH-adjusted SD selective medium for 24 h. The GFP fluorescence of 10,000 cells was measured by flow cytometry. Mean values of the green fluorescence signal of 10,000 cells. The solid lines represent the mean value for the positive control and the negative control (996 and 160 respectively). The dashed lines marks the 3 standard deviation cut off for both the positive and negative control (standard deviation of 112 and 5.6 respectively). Z’ factor > 0.5 indicates assays robust and suitable for screening.
(TIF)

Figure S2 **Plasmids used in this study.** (A) Single-copy plasmid pGK416-ZsGreen (B) Multi-copy plasmid pGK426-NTS42 (C) Integration plasmid pBlue-FIG1pt-ZsGreen (D) Integration plasmid pBlue-HIS3pt-FIG1pt-ZsGreen.
(TIF)

Table S1 **List of oligonucleotides.**
(PDF)
