# Supplementary figures and images for: Bright Fluorescence Monitoring System Utilizing Zoanthus sp. Green Fluorescent Protein (ZsGreen) for Human G-Protein-Coupled Receptor Signaling in Microbial Yeast Cells
Source: PLoS One. 2013 Dec 5;8(12):e82237. doi: 10.1371/journal.pone.0082237 (PMC3855394; doi:10.1371/journal.pone.0082237)

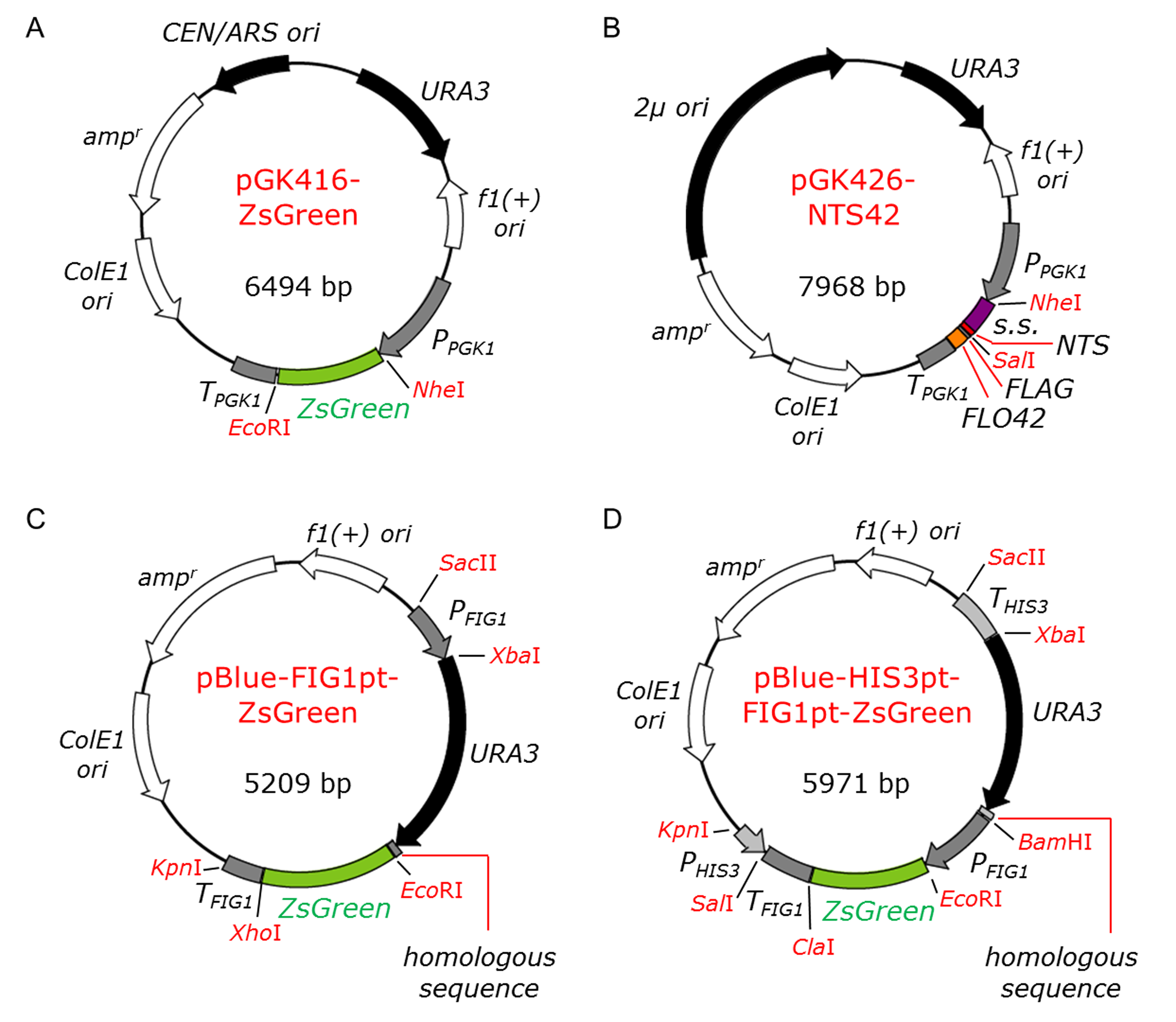

Supplement: Figure S2 — Plasmids used in this study. (A) Single-copy plasmid pGK416-ZsGreen (B) Multi-copy plasmid pGK426-NTS42 (C) Integration plasmid pBlue-FIG1pt-ZsGreen (D) Integration plasmid pBlue-HIS3pt-FIG1pt-ZsGreen. (TIF) [file pone.0082237.s002.tif]
