# Supplementary material for: Bright Fluorescence Monitoring System Utilizing Zoanthus sp. Green Fluorescent Protein (ZsGreen) for Human G-Protein-Coupled Receptor Signaling in Microbial Yeast Cells
Source: PLoS One. 2013 Dec 5;8(12):e82237. doi: 10.1371/journal.pone.0082237 (PMC3855394; doi:10.1371/journal.pone.0082237)
Supplement: Table S1 — List of oligonucleotides. (PDF) [file pone.0082237.s003.pdf]

PLoS ONE
Supporting Information:

Bright fluorescence monitoring system utilizing *Zoanthus* sp. green fluorescent protein (*ZsGreen*) for human G-protein-coupled receptor signaling in microbial yeast cells

Table S1. List of oligonucleotides.

o#	Name	Sequence
o1	NheI_ZsGreen_fw	5'-TTTTGCTAGCATGGCTCAGTCAAAGCACGG
o2	EcoRI_ZsGreen_rv	5'-TTTTGAATTCTCAGGGCAATGCAGATCCGG
o3	SacII_dFIG1up_fw	5'-GGGGCCGCGGTACAAAAATTATAACATTTT
o4	XbaI_dFIG1up_rv	5'-CCCCTCTAGATTTTTTTTTTTTTTTTTTGT
o5	XbaI_URA3_fw	5'-CCCCTCTAGATTTTTTGTTCTTTTTTTTGA
o6	EcoRI_hr40-URA3_rv	5'-CCCCGAATTCTTTTTTTTTTTTTTTTTTGTTTGTTTGTTTGTTTGTTTACGGGTA ATAACTGATATAATT
o7	EcoRI_ZsGreen_fw	5'-TTTTGAATTCATGGCTCAGTCAAAGCACGG
o8	XhoI_ZsGreen_rv	5'-TTTTCTCGAGTCAGGGCAATGCAGATCCGG
o9	XhoI_dFIG1dn_fw	5'-GGGGCTCGAGTTTTATCCTCAAATAAACAT
o10	KpnI_dFIG1dn_rv	5'-CCCCGGTACCAACAGACGGTAATGATTAGA
o11	SacII_dHIS3dn_fw	5'-AAAACCGCGGTATGAAATGCTTTTCTTGTT
o12	XbaI_dHIS3dn_rv	5'-GGGGTCTAGATGACACCGATTATTTAAAGC
o13	BamHI_hr40-URA3_rv	5'-CCCCGGATCCTGACACCGATTATTTAAAGCTGCAGCATACGATATATATAGGGT AATAACTGATATAATT
o14	BamHI_PFIG1_fw	5'-AAAAGGATCCATCACCCTGCATTGCCTCTT
o15	EcoRI_PFIG1_rv	5'-CCCCGAATTCTTTTTTTTTTTTTTTTTTGTTTGTTTGTTT
o16	ClaI_ZsGreen_rv	5'-TTTTATCGATTCAGGGCAATGCAGATCCGG
o17	ClaI_TFIG1_fw	5'-GGGGATCGATTTTTATCCTCAAATAAACAT
o18	SalI_TFIG1_rv	5'-CCCCGTCGACATAACATTAGTATTTATAAA
o19	SalI_dHIS3up_fw	5'-AAAAGTCGACCTTTGCCTTCGTTTATCTTG
o20	KpnI_dHIS3up_rv	5'-AAAAGGTACCTCTTGGCCTCCTCTAGTACA
o21	NheI_SSTR2_fw	5'-TTTTGCTAGCATGGACATGGCGGATGAGCC
o22	BglII_SSTR2_rv	5'-CCCCAGATCTTCAGATACTGGTTTGGAGGT
o23	NheI_NTSR1_fw	5'-AAAAGCTAGCATGCGCCTCAACAGCTCCGC
o24	BglII_NTSR1_rv	5'-CCCCAGATCTCTAGTACAGCGTCTCGCGGG
o25	NheI_SS_fw	5'-GGGGGCTAGCATGAGATTTCCTTCAATTTT
o26	NTS_SS_rv	5'-ATTCTCATACAGCTGTCTTTTATCCAAAGA
o27	SalI_NTS_rv	5'-AAAAGTCGACGAGTATGTAGGGTCTTCTGGGTTTATTCTCATACAGCTG
o28	SalI_NTS(8-13)_rv	5'-GGGGGTCGACGAGTATGTAGGGTCTTCTTCTTTTATCCAAAGATACCC
o29	SalI_NMN_rv	5'-GGGGGTCGACCAGAATATAAGGAATTTTTCTTTTATCCAAAGATACCC
